# Interleukin-6 and Interleukin-8 Regulate STAT3 Activation Migration/Invasion and EMT in Chrysophanol-Treated Oral Cancer Cell Lines

**DOI:** 10.3390/life11050423

**Published:** 2021-05-05

**Authors:** Po-Chih Hsu, Yi-Hsuan Chen, Ching-Feng Cheng, Chan-Yen Kuo, Huey-Kang Sytwu

**Affiliations:** 1 National Defense Medical Center, Graduate Institute of Medical Sciences, Taipei 114, Taiwan; pino0906@gmail.com; 2 Department of Dentistry, Taipei Tzu Chi Hospital, Buddhist Tzu Chi Medical Foundation, New Taipei City 231, Taiwan; lucy199043@gmail.com; 3 Department of Pediatrics, Taipei Tzu Chi Hospital, Buddhist Tzu Chi Medical Foundation, Taipei 114, Taiwan; chengcf@mail.tcu.edu.tw; 4 Institute of Biomedical Sciences, Academia Sinica, Taipei 114, Taiwan; 5 Department of Pediatrics, Tzu Chi University, Hualien 970, Taiwan; 6 Department of Research, Taipei Tzu Chi Hospital, Buddhist Tzu Chi Medical Foundation, New Taipei City 231, Taiwan; 7 National Institute of Infectious Diseases and Vaccinology, National Health Research Institutes, Zhunan 350, Taiwan; 8 Department of Microbiology and Immunology, National Defense Medical Center, Taipei 114, Taiwan

**Keywords:** chrysophanol, IL-6, IL-8, EMT, migration, invasion, EMT, STAT3

## Abstract

The tumor microenvironment plays a critical role in the control of metastasis. The epithelial–mesenchymal transition (EMT) is strongly associated with tumor metastasis, and consists of several protein markers, including E-cadherin and vimentin. We discovered that chrysophanol causes oral cancer cell apoptosis and the inhibition of migration/invasion and EMT. However, the detailed mechanisms of chrysophanol and its role in oral cancer with respect to the tumor microenvironment remain unknown. In the clinic, proinflammatory cytokines, such as IL-6 and IL-8, exhibit a higher expression in patients with oral cancer. However, the effect of chrysophanol on the production of IL-6 and IL-8 is unknown. We evaluated the expression of IL-6 and IL-8 in human SAS and FaDu oral cancer cell lines in the presence or absence of chrysophanol. The migration and invasion abilities were also determined using a Boyden chamber assay. Our results showed that treatment with chrysophanol significantly decreased the expression of IL-6 and IL-8, as well as the invasion ability of oral cancer cells. Moreover, chrysophanol also attenuated the EMT by increasing the expression of E-cadherin and reducing the expression of vimentin. Mechanistically, chrysophanol inhibited IL-6- and IL-8-induced invasion and STAT3 phosphorylation. IL-6 and IL-8 promote EMT and cell invasion, which is potentially related to the STAT3 signaling pathway in oral cancer. These findings provide insight into new aspects of chrysophanol activity and may contribute to the development of new therapeutic strategies for oral cancer.

## 1. Introduction

Oral cancer is a significant global health problem [1]. The most common type of oral cancer is squamous cell carcinoma [2]. It has been reported that, alongside the cancerous epithelial cells themselves, the tumor microenvironment (TME) impacts the progression of head and neck squamous cell carcinoma (HNSCC). The TME consists of cancer-associated fibroblasts, immune cells, and other supporting cells [3]. Interestingly, the tumor cell secretome (TCS) is secreted from tumors and prevents chemotherapy-dependent cytotoxicity [4,5]. The TCS includes, but is not limited to, cytokines, growth factors, and enzymes [5] that regulate cell survival, proliferation, metastasis, angiogenesis, and differentiation, and also promote interactions between cells [4]. It is becoming clear that studies on the differences in the stromal composition of the HNSCC tumor microenvironment and its impact on cancer development and progression may improve our understanding of the mechanisms underlying the different responses to therapy and allow us to identify new targets for clinical intervention [6]. Clinical studies involving conventional modalities and first-line agents, including epidermal growth factor receptor (EGFR) inhibitors, monoclonal antibodies (mAbs), and tyrosine kinase inhibitors, have been conducted for many years. These interventions target overexpressed EGFR, which are detected in up to 80% of HNSCCs [7,8,9,10]. However, interactions of the tumor microenvironment (TME) influence tumor progression and treatment response [11]. As a result, strategies for treating HNSCC have had a limited impact on progression-free survival and overall survival. Therefore, it is important to develop new methods for reducing the TCS [5].

Cytokines are released constantly from HNSCC, including interleukin-6 (IL-6) and interleukin-8 (IL-8) [12]. Both IL-6 and IL-8 belong to the interleukin family (IL), act as proinflammatory cytokines, and further activate downstream pathways through their receptors, IL-6 receptor (IL-6R) and IL-8 receptor (IL-8R). IL-6 functions in (1) proliferation through RAS/RAF/MAPK, Akt/PI3K/mTOR, STATs, and Src/YAP/Notch; (2) survival, metabolism and oxidative stress through Akt/PI3K/mTOR and STAT signaling; and (3) in tissue generation through Src/YAP/Notch signaling. Evidence also indicates it has a role in regulating migration, angiogenesis, differentiation, and immune regulation. STATs also play a role in these tumor progression processes [13]. IL-8 has been well-characterized in tumorigenesis and has been shown to induce not only proliferation but also blood vessel angiogenesis, invasion, chemotaxis, and apoptosis regulation through STAT3/NF-κB in several cancer types [14,15,16]. Both IL-6 and IL-8 can induce chemotactic activities and direct neutrophils and monocytes to inflammatory tissues [17,18], and both the IL-6/STAT3 and IL-8/STAT3 axes mediate malignancies, cancer stemness, and immune suppression [14,19]. Furthermore, studies have shown that cross-talk between the STAT3 and EGFR pathways attenuates the effects of standard therapies and EGFR-targeting treatments on cancer [20,21,22]. Therefore, it is important to develop strategies for reducing proinflammatory cytokines when administering cancer treatments.

Chrysophanol is an anthraquinone derivative that is also known as 1,8-dihydroxy-3-methyl-anthraquinone. It was first identified in an extract from *Rheum rhabarbarum* [23]. It has multiple pharmacological effects, including antioxidation, anti-inflammatory [24], antiviral, antibacterial, and anticancer activity, in various cancers, including colorectal, breast, and oral cancer [25,26,27]. Park et al. demonstrated that chrysophanol inhibited breast cancer cell growth by regulating endoplasmic reticulum stress and reactive oxygen species production (ROS) through the AKT and mitogen-activated protein kinase (MAPK) signaling pathways [28]. In previous studies, we showed that chrysophanol significantly induced cell death by apoptosis through ROS accumulation. Moreover, chrysophanol caused cell cycle arrest and inhibited migration/metastasis through the epithelial–mesenchymal transition (EMT) and Wnt-3-dependent pathways [27,29]. However, the effect of chrysophanol on the inflammatory response in metastasis in oral cancer cell is unclear. Other studies have reported decreased concentrations of IL-6 in chrysophanol-treated cultural medium [23,24]. The effect of the TCS following chrysophanol treatment and evaluation of its impact in terms of regulating interactions between the tumor microenvironment, tumor cells, and immune cells are important issues. Thus, the aim of this study was to investigate whether chrysophanol attenuates migration/invasion and EMT by regulating IL-6 and IL-8 in HNSCC cell lines.

## 2. Materials and Methods

### 2.1. Reagents

Chrysophanol (CAS 481-74-3) and N-acetyl-L-cysteine (CAS 616-91-1) were obtained from Cayman Chemical (Ann Arbor, MI, USA). Dulbecco’s Modified Eagle Medium and the Pierce BCA Protein Assay Kit (#23225) were obtained from ThermoFisher Scientific (Waltham, MA, USA). Fetal bovine serum was purchased from Corning (Corning, NY, USA). Penicillin/streptomycin was obtained from Bioindustry (London, UK). PhosSTOP and complete ULTRA Tablets were purchased from Roche (Basel, Switzerland). The Human Cytokine Array Kit was provided by R&D Systems (#ARY005B, Minneapolis, MN, USA). Anti-IL-6 (#12153), E-cadherin (#3195), CD44 (#5640), and PD-L1 (#13684) antibodies were purchased from Cell Signaling (Danvers, MA, USA). Antivimentin was obtained from BioLegend (San Diego, CA, USA). Anti-STAT3 (A11216), phosphor-STAT3 (AP0136), IL-8 (A2541), and actin (AC026) antibodies were purchased from ABclonal (Woburn, MA, USA). Human Recombinant Interleukin-6 (#I1359) and IL-8 (CXCL8) (#SPR3098) were purchased from Sigma-Aldrich (St. Louis, SL, USA). Human IL-6 (#D6050) and IL-8/CXCL8 (#D8000C), as well as the Quantikine ELISA Kit, were purchased from R&D Systems (Minneapolis, MN, USA).

### 2.2. Cell Culture

FaDu (human pharynx squamous cell carcinoma) and SAS (human tongue squamous carcinoma) cell lines were obtained from the ATCC and National Defense Medical Center, respectively. The cells were analyzed for mycoplasma and tested negative. Cell lines were cultured in Dulbecco’s Modified Eagle Medium, containing 10% fetal bovine serum and 1% penicillin/streptomycin, and incubated in a 5% CO₂ atmosphere at 37 °C.

### 2.3. Cytokine Array

The cytokine array analysis was performed according to the method of a previous study [30], with some modifications. Cells were untreated (control) or treated with chrysophanol, and the medium was replaced with serum- and antibiotic-free DMEM for the final 24 h of the incubation. Medium from three wells was pooled, clarified by centrifugation at 200× *g* at 4 °C, and immediately applied to the Human Cytokine Array Kit according to the manufacturer’s instructions (R&D Systems, Minneapolis, MN, USA). The cytokine array signal was detected at multiple exposure times, ranging from 15 s to 10 min. The film was scanned using a ChemiDocTMXRS + System (Bio-Rad Laboratories, Hercules, CA, USA). Signal levels were measured using ImageJ software, with the Protein Array Analyzer plugin 16.

The Human Cytokine Array Kit was provided by R&D Systems and was used according to manufacturer’s introduction and the method suggested by a previous study [31]. The image of spots was scanned and measured using NIH ImageJ software with the Protein Array Analyzer plugin 16.

### 2.4. Western Blot Analysis

SDS-PAGE was performed using 10% or 12% acrylamide gels, with equal amounts (30 μg) of protein loaded per lane. After electrophoresis, the proteins were transferred to polyvinylidene fluoride (PVDF) membranes at 350 mA for 2 h. For blocking, the membranes were soaked in 5% nonfat milk at room temperature for 1 h at 75 rpm. Then, the membrane was incubated with primary antibodies at 4 °C overnight. The following day, the membranes were washed three times with TBS buffer containing 0.2% Tween 20 (Bionovas, Halifax, Canada) at room temperature for 10 min each. Then, incubation was completed with secondary antibody conjugated with horseradish peroxidase (HRP) at a 1:10,000 dilution for 1 h at room temperature. After washing in TBS buffer with 0.2% Tween 20, the Western HRP substrate (LuminataTM Classico, Millipore, Darmstadt, Germany) was used to develop the fluorescent signal, which was visualized with a ChemiDoc^TM^XRS + System (Bio-Rad Laboratories, Hercules, CA, USA).

### 2.5. Cell Invasion Assay

The cell invasion assay was performed using 24-well plates (pore size 8 μm; PI8P01250, Millipore) according to the manufacturer’s instructions, and the method suggested by a previous study [32]. Briefly, cells were starved in serum-free DMEM medium for 12 h. Then, 5.0 × 10^4^ cells were seeded into the upper chamber, which was coated with Matrigel (#356234, BD Bioscience, CA, USA). Complete medium was added to the lower chamber. After a 24 h incubation, the cells were stained with 0.1% crystal violet. The cells that had invaded through the Matrigel were counted under a microscope in five predetermined fields (×200). Experiments were carried out in triplicate.

### 2.6. Statistical Analysis

Data are expressed as the mean ± standard error of the mean, and statistical comparisons were calculated by one-way or two-way analysis of variance (ANOVA), followed by a Bonferroni post hoc test. A value of *p* < 0.05 was considered statistically significant.

## 3. Results

### 3.1. Effect of Chrysophanol on the Expression of Inflammatory Cytokines

**Figure 1:**
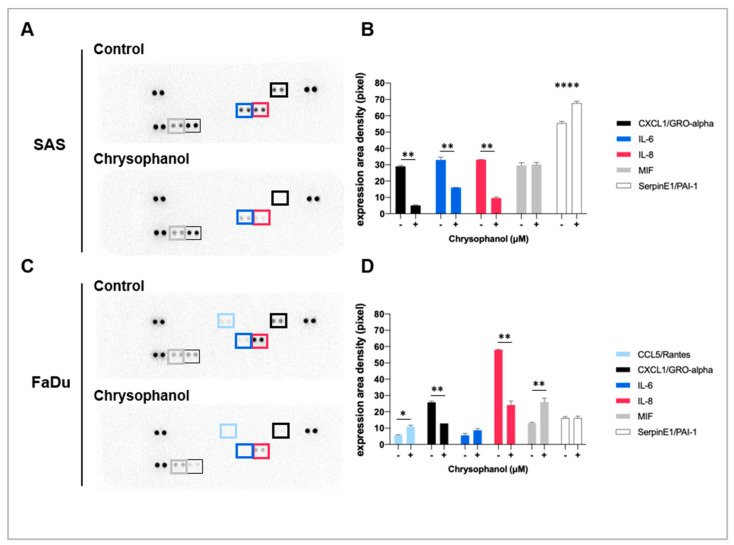
The effects of chrysophanol on inflammatory cytokines in SAS and FaDu cell lines. (**A**) The panel shows an image of the signal spots on the membrane for each cytokine. Each spot represents one cytokine, and each cytokine is spotted in duplicate. Spots that show significant changes are marked in the SAS and FaDu cell lines. (**B**) The image of the spots was scanned and measured using NIH ImageJ software. The relative expression level of each protein was calculated from densitometry data from A and normalized to the control group (in the absence of chrysophanol). (**C**) The panel shows an image of the signal spots on the membrane for each cytokine. Each spot represents one cytokine and each cytokine is represented in duplicate. Spots that show significant changes are marked in the FaDu cell line. (**D**) The image of spots was scanned and measured using NIH ImageJ software. The relative expression level of each protein was calculated from densitometry data from C and normalized to the control group (in the absence of chrysophanol). Error bars indicate SD (*n* = 2). * *p* < 0.05. ** *p* < 0.01. **** *p* < 0.001.

Our previous study showed that chrysophanol ameliorated EMT in oral cancer cells through a Wnt-3-dependent pathway [27] and also regulated oral cancer cell death, ROS production, and metastasis [29]. Among the 36 tested cytokines, chrysophanol (12.6 μM in SAS and 9.64 μM in FaDu) downregulated the production of two cytokines, CXCL1 (C-X-C Motif Chemokine Ligand-1) and IL-8, in both SAS and FaDu cell lines (Figure 1), but only IL-6 in SAS cells (Figure 1B,D). In contrast, chrysophanol upregulated the production of Serpin E1/PIA-1 (Plasminogen Activator Inhibitor-1) in SAS cells (Figure 1A,B), as well as CCL5 (Rantes) and MIF (Macrophage Migration Inhibitory Factor) in FaDu cells (Figure 1C,D). It has been reported that proinflammatory cytokines, such as IL-6 and IL-8, are important mediators of the inflammatory response [33], and the protein levels of these cytokines were suppressed by chrysophanol (Figure 1). Thus, the results suggest that chrysophanol reduces the inflammatory response through specific pathways.

### 3.2. Chrysophanol Alleviates Inflammation and EMT and May Improve Antitumor Immunity in HNSCC Cell Lines

**Figure 2:**
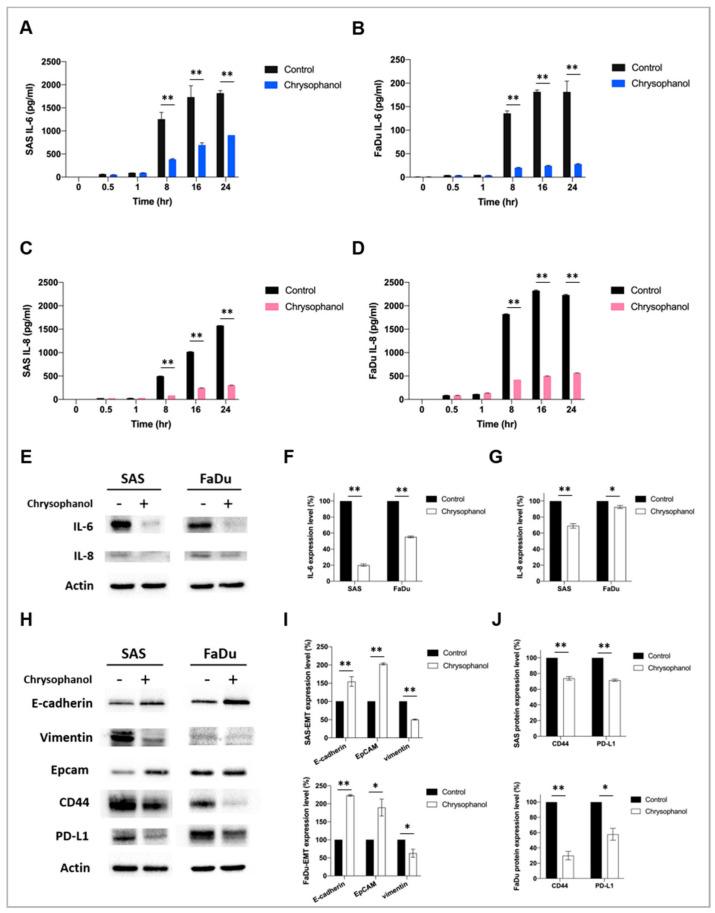
Effect of chrysophanol on inflammation and EMT in SAS and FaDu cell lines. (**A**,**B**) IL-6 and (**C**,**D**) IL-8 ELISA results of SAS and FaDu cell conditioned medium after treatment with chrysophanol (12.6 μM in SAS and 9.64 μM in FaDu) (29) or without (control) at 0, 0.5, 1, 8, 16, and 24 h. (**E**) Changes in the expression of IL-6 and IL-8. Actin was used as an internal control. (**F**,**G**) Quantitative results showing the level of specific proteins assessed by ImageJ. (**H**) Changes in the expression of E-cadherin, vimentin, CD44, and PD-L1. Actin was used as an internal control. (**I**,**J**) Quantitative results showing the level of specific proteins assessed by ImageJ. All data are presented as the mean ± SD. *n* = 3. * *p* < 0.05. ** *p* < 0.01. Whole blot is included in Figure S1 in Supplementary materials.

To further confirm the effect of chrysophanol on the production of IL-6 and IL-8 in conditioned medium, we measured IL-6 and IL-8 using ELISA. The results showed that chrysophanol significantly reduced the production of IL-6 in both SAS (Figure 2A) and FaDu cell lines (Figure 2B), as well as IL-8 in both SAS (Figure 2C) and FaDu cell lines (Figure 2D), in a time-dependent manner. The results showed that chrysophanol significantly downregulated the expression of IL-6 in both SAS and FaDu cell lines (Figure 2E–G). We also evaluated the expression of the EMT markers, E-cadherin and vimentin [34]. The results indicated that chrysophanol upregulated the expression of E-cadherin but downregulated the expression of vimentin in both SAS and FaDu cell lines (Figure 2H,I). Chrysophanol also downregulated the expression of CD44 and PD-L1 in both SAS and FaDu cell lines (Figure 2H–J). Taken together, we suggest that chrysophanol alleviates inflammation and EMT, and it may contribute to antitumor immunity.

### 3.3. Chrysophanol Inhibits SAS Cell Invasion and STAT3 Activation in an IL-6- and IL-8-Dependent Manner

**Figure 3:**
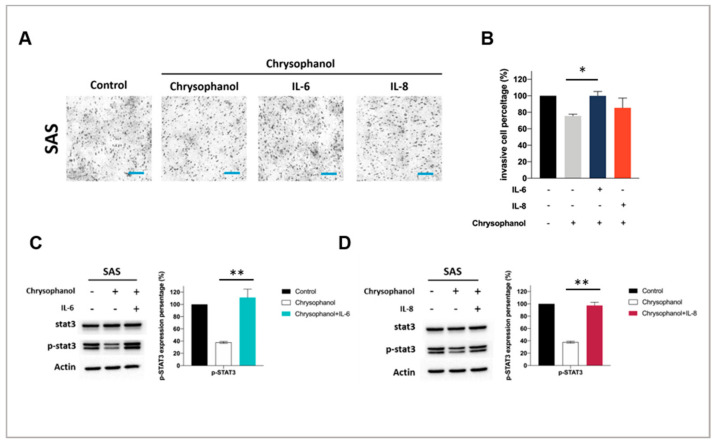
Chrysophanol inhibits invasion and STAT3 phosphorylation in SAS cells. (**A**) After 24 h exposure of SAS cell to chrysophanol, the invasion assay was performed. (**B**) Experiments were performed in triplicate. *n* = 3. ** *p* < 0.01. (**C**,**D**) Changes in the expression of phospho-Stat3 in the presence of IL-6 (**C**) and IL-8 (**D**), with or without chrysophanol treatment. Actin was used as an internal control. Quantitative results showing the level of specific proteins assessed by ImageJ. All data are presented as the mean ± SD. *n* = 3. Scale bar = 100 μm. * *p* < 0.05. ** *p* < 0.01. Whole blot is included in Figure S2 in Supplementary materials.

To further elucidate whether chrysophanol has an anti-invasion effect in an IL-6- and IL-8-dependent manner, we utilized a Matrigel invasion assay. The results showed that chrysophanol (12.6 μM in SAS) significantly decreased SAS cell invasion, but this effect was restored after IL-6 (100 ng/mL) and IL-8 (100 ng/mL) treatment (Figure 3A,B). As shown in Figure 3C,D, our data indicate that chrysophanol downregulated the expression of phospho-STAT3, but the downregulation was reversed in the presence of IL-6 and IL-8. Therefore, chrysophanol prevented IL-6- and IL-8-induced phosphorylation of STAT3, which may provide a competitive environment for OSCC cell invasion.

## 4. Discussion

Our in vitro study showed that chrysophanol reduced inflammatory responses, epithelial–mesenchymal transition, and invasion of oral cancer cell lines. Its main effects were mediated by a reduction in IL-6 and IL-8 expression and a consequent decrease in STAT3 phosphorylation, as well as an increase in E-cadherin and decrease in vimentin expression. The focus of our study was primarily on inhibiting migration and EMT; however, there are also other promising agents, such as gramine, which could play a role in inhibiting the process of oral carcinogenesis [35]. Further studies with various natural extracts and synthetic compounds are needed to find optimal treatment strategies for HNSCC, especially when conventional approaches fail. The main clinical implication of our findings is that chrysophanol may be considered in order to reduce the risk of metastasis in patients with oral cancer, considering that chrysophanol was shown to reduce the invasion ability of tumor cells and reduce epithelial–mesenchymal transition.

**Figure 4:**
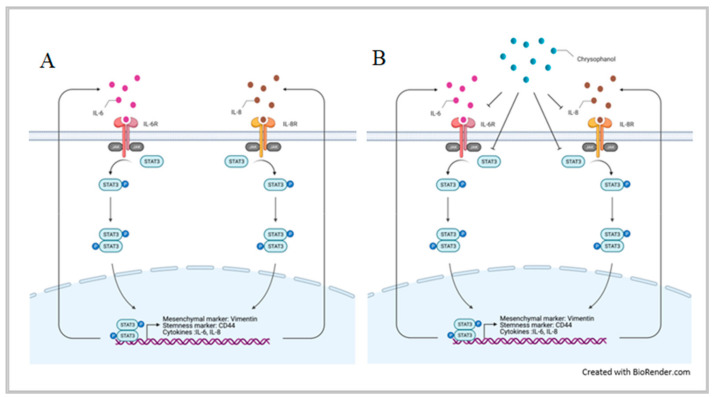
Schematic representation of the anti-EMT and invasion mechanism of chrysophanol via IL-6 and IL-8 regulation. (**A**) The production of IL-6 and IL-8 induces STAT3 phosphorylation and increases nuclear p-STAT3 translocation, thus further regulating the expression of its downstream target genes, which promote oral cancer EMT and invasion. (**B**) Chrysophanol downregulates IL-6 and IL-8 and abrogates the function of p-STAT3 by decreasing its level in the nucleus, thus suppressing EMT and invasion. This figure was created with BioRender.com.

As shown in Figure 1 and Figure 2A–G, our results indicated that chrysophanol reduced IL-6 and IL-8 expression in conditioned medium and cell lysates. IL-6 and IL-8 are proinflammatory cytokines released from HNSCC [12], with possible roles in tumor migration, angiogenesis, and immunity. Indeed, several studies have indicated that EMT is mediated by changes in the TME [35,36]. IL-6 and IL-8 have been shown to cause immunosuppression in the TME [37,38] and regulate tumor progression, metastasis, and invasion [39,40]; in this context, immune-based therapeutic intervention targeting IL-6 and IL-8 may represent a new therapeutic strategy in patients with oral cancer. Consistent with our findings, Fu and Lin reported that inhibiting IL-6 and IL-8 signaling using novel drug combinations (e.g., bazedoxifene/reparixin, bazedoxifene/SCH527123) achieved more effective treatment of triple-negative breast cancer and pancreatic ductal adenocarcinoma [41]. Simvastatin was shown to inhibit the release of IL-6 and IL-8 from colorectal cell lines [42] and in patients with rheumatoid arthritis [43]. Considering the clinical data showing that IL-6 and IL-8 may increase the pathogenicity of HNSCC, these cytokines may be useful biomarkers or targets for therapy [44]. Indeed, our in vitro findings showed that chrysophanol reduced the invasion ability of oral cancer cell lines by inhibiting the expression of IL-6 and IL-8. The effect was mediated by chrysophanol-promoted blockade of IL-6- and IL-8-induced phosphorylation of STAT3 (Figure 4). The relationship between IL-6 and STAT3 phosphorylation was also supported by a recent study that showed that IL-6 increased the migration properties of oral squamous cancer cells in a manner dependent on the STAT3 phosphorylation level [45]. These results provide further experimental support for potential targeted treatments of oral cancer based on exploiting the molecular basis of oral cancer development via migration/invasion and EMT.

Invasion and metastasis are the key indicators of a poor prognosis [45,46,47,48,49,50], and they are based on the EMT. Consistent with our previous findings [29], here we showed that chrysophanol attenuated the EMT by upregulating E-cadherin and downregulating vimentin in both the SAS and FaDu cell lines (Figure 2H–J). Likewise, chrysophanol also inhibited SAS cell invasion ability (Figure 3), but it was reversed by IL-6 and IL-8 treatment (Figure 3A,B), further confirming that the effects were IL-6- and IL-8-dependent. Previous studies have reported that regulation of oral cancer metastasis involves many signaling pathways, such as MAPK, PI3K/AKT, and FAK/Src [51,52]. Recently, IL-6 has been shown to stimulate migration of oral cancer cells via the phosphorylation of STAT3 [45]. In this context, our results showed that chrysophanol decreased the expression of phospho-STAT3 in SAS cells (Figure 3C,D), which was restored after IL-6 and IL-8 treatment (Figure 3C,D), confirming that the effects of chrysophanol on invasion ability are IL-6- and IL-8-dependent (Figure 4). Our findings are consistent with those of a previous study that showed that IL-6 promoted HNSCC migration/invasion and EMT by triggering EMT through downregulating E-cadherin and STAT3 signaling [53]. Similar effects have been found in other cancers—namely, IL-6 has been shown to induce the activation of the EMT program via STAT3 activation in breast cancer [54] and to play a role in cancer stemness of osteosarcoma cells via STAT3 activation [55]. Xu et al. reported that IL-8 promoted the malignant progression of HNSCC cells via STAT3 phosphorylation [56]. Therefore, the induction of EMT through the STAT3 signaling pathway, mediated by IL-6 and IL-8, is important to the metastasis of HNSCC, and is associated with poor clinical outcomes [32].

Tumor cells escape immune surveillance through CD44-dependent upregulation of programmed death ligand 1 (PD-L1) [57]. Here, we showed that chrysophanol may contribute to abolishing or attenuating immune escape by downregulating the expression of CD44 and PD-L1 (Figure 2H,J). Considering that chrysophanol also inhibited STAT3, there was obviously an association between STAT3 and CD44/PD-L1. Indeed, a previous study showed that inhibiting the phosphorylation of STAT3 effectively inhibited the expression of PD-L1 in oral cancer cell lines (CAL27 and FaDu) and *Tgfbr1/Pten* 2cKO, an HNSCC mouse model [58].

The main limitation of our study is that it was fully based on in vitro assessment of two cell lines; in vivo studies and additional experimental in vitro studies are needed to confirm our findings and provide further clinically relevant information. Specifically, in our study, treatment with IL-6 and IL-8 reversed the effects of concomitant chrysophanol treatment—i.e., it restored the invasion ability and the p-STAT3 levels, indicating that the invasion ability and phosphorylation of p-STAT3 are IL-6- and IL-8-dependent processes. However, direct targeting of IL-6 and IL-8 using neutralizing antibodies and transient silencing, or pharmacological inhibition of STAT3, may be useful for providing additional confirmation of our findings. In addition, our study showed that chrysophanol reduced the expression of CD44 and PD-L1, which may affect immunotherapy. However, the modulation of PD-L1 expression in cell lines is just the first step in evaluating whether chrysophanol may influence immunotherapy in the clinical context; therefore, experimental animal studies should be combined with in vitro studies to further understand the relevance of altered PD-L1 expression.

## 5. Conclusions

In conclusion, our findings suggest that overexpression of IL-6 and IL-8 promotes the EMT and invasion abilities of oral cancer cells through the STAT3 signaling pathway and also decreases E-cadherin expression and increases vimentin expression; these effects are abolished by chrysophanol. Therefore, chrysophanol has the potential to inhibit migration/invasion and EMT through the inhibition of IL-6- and IL-8-induced activation of STAT3 in oral cancer (Figure 4).

## Data Availability

The data presented in this study are available on request from the corresponding author.

## Supplementary Materials

The following are available online at https://www.mdpi.com/article/10.3390/life11050423/s1, Figure S1: Effect of chrysophanol on inflammation and EMT in SAS and FaDu cell lines—whole blot, Figure S2: Chrysophanol inhibits invasion and STAT3 phosphorylation in SAS cells—whole blot.
