# Supplementary material for: Interleukin-6 and Interleukin-8 Regulate STAT3 Activation Migration/Invasion and EMT in Chrysophanol-Treated Oral Cancer Cell Lines
Source: Life (Basel). 2021 May 5;11(5):423. doi: 10.3390/life11050423 (PMC8148210; doi:10.3390/life11050423)
Supplement: Supplementary file 1 [file life-11-00423-s001.zip › life-1185350-supplementary.pdf]

Supplementary materials:

Interleukin-6 and Interleukin-8 Regulate STAT3 Activation Migration/Invasion and EMT in Chrysophanol-Treated Oral Cancer Cell Lines

Po-Chih Hsu [1,2], Yi-Hsuan Chen [2], Ching-Feng Cheng [3,4,5], Chan-Yen Kuo [6,*], Huey-Kang Sytwu [7,8,*]

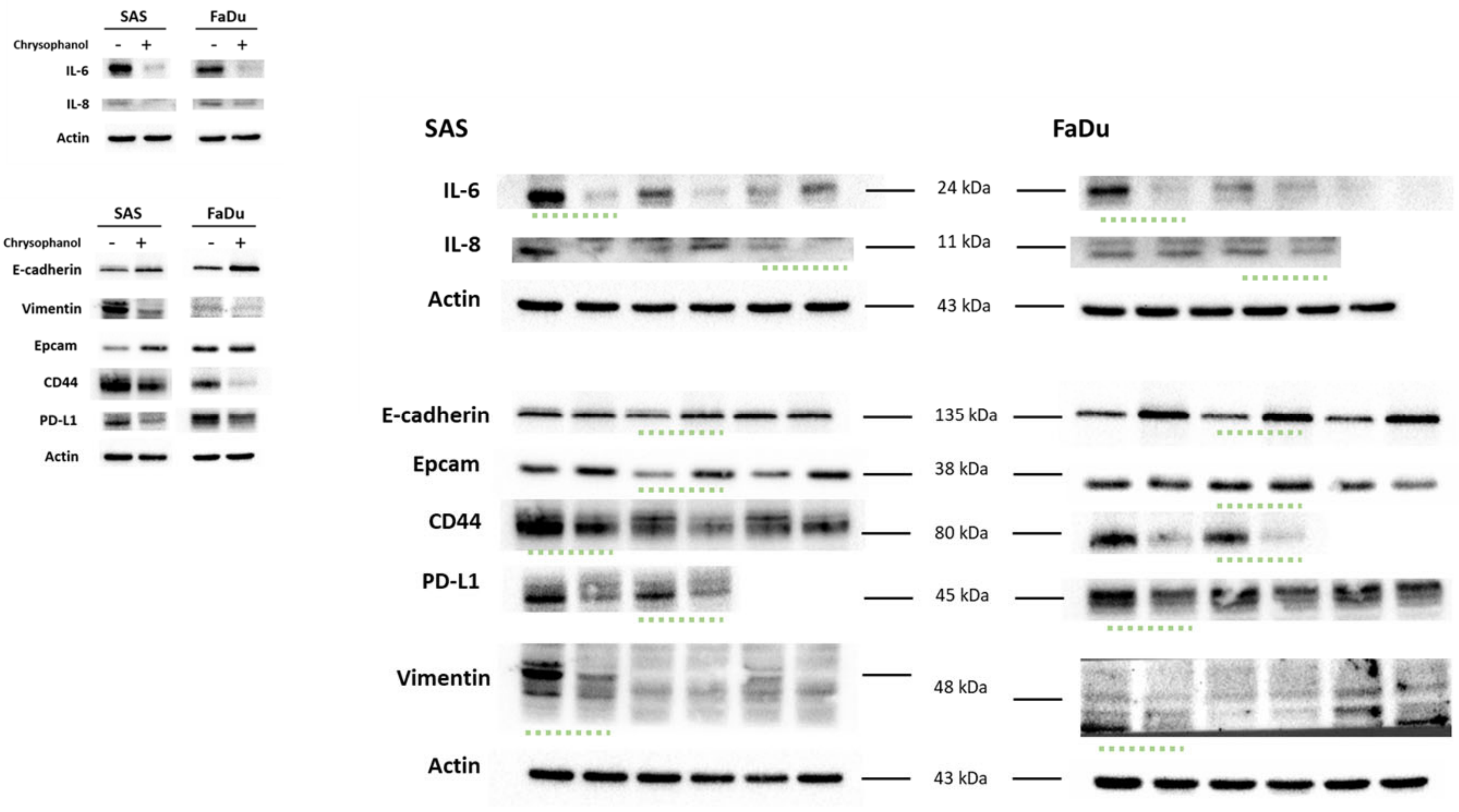

Figure S1. Effect of chrysophanol on inflammation and EMT in SAS and FaDu cell lines – whole blot.

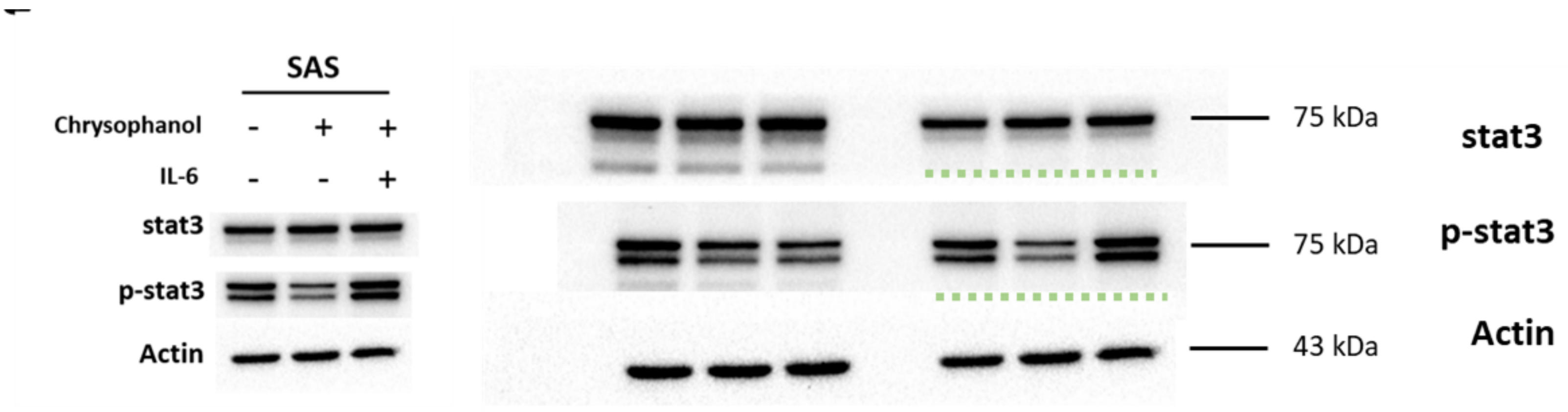
SAS
Chrysophanol - + +
IL-6 - - +
stat3
p-stat3
Actin
75 kDa
stat3
75 kDa
p-stat3
43 kDa
Actin

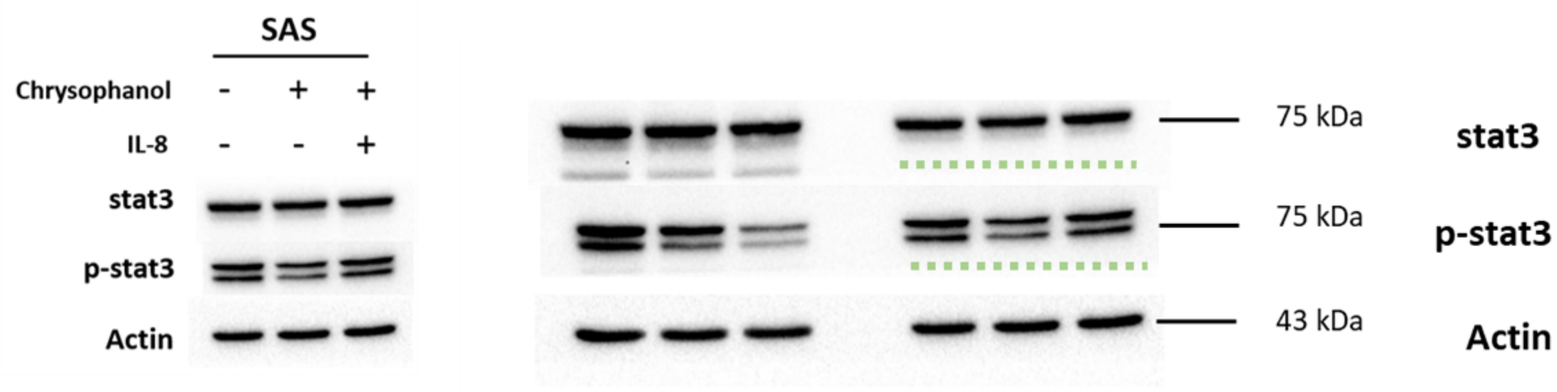
SAS
Chrysophanol - + +
IL-8 - - +
stat3
p-stat3
Actin
75 kDa
stat3
75 kDa
p-stat3
43 kDa
Actin

Figure S2: Chrysophanol inhibits invasion and STAT3 phosphorylation in SAS cells – whole blot.

**Figure S1**		Control				Chrysophanol-SAS				Chrysophanol-FaDu						
	IL-6	100	100	100		19	21	18.2		54.5	56	54.7				
	IL-8	100	100	100		71	67	65.2		94	91.3	92.7				
	E-cadheri	100	100	100		164	145	141		222	225	220				
	EpCAM	100	100	100		201	205	182.4		206.2	172.6	198				
	Vimentin	100	100	100		51.1	49.3	53.9		55.1	70.71	59				
	CD44	100	100	100		75.5	72.1	70		33.9	26	31.5				
	PD-L1	100	100	100		70.4	72.5	70.1		52.36	63.3	55				
**Figure S2**		Control				Chrysophanol-SAS				IL-6-SAS				IL-8-SAS		
	p-STAT3	100	100	100		36.8	39.1	40.2		101.6	120.9	117.7		101.1	93.8	89.3
		100	100	100		38.37	35.4									
